# Regional Assessment of Human Fecal Contamination in Southern California Coastal Drainages

**DOI:** 10.3390/ijerph14080874

**Published:** 2017-08-04

**Authors:** Yiping Cao, Meredith R. Raith, Paul D. Smith, John F. Griffith, Stephen B. Weisberg, Alexander Schriewer, Andrew Sheldon, Chris Crompton, Geremew G. Amenu, Jason Gregory, Joe Guzman, Kelly D. Goodwin, Laila Othman, Mayela Manasjan, Samuel Choi, Shana Rapoport, Syreeta Steele, Tommy Nguyen, Xueyuan Yu

**Affiliations:** 1 Southern California Coastal Water Research Project, 3535 Harbor Blvd, Suite 110, Costa Mesa, CA 92626, USA; mraith@sccwrp.org (M.R.R.); pauls@sccwrp.org (P.D.S.); johng@sccwrp.org (J.F.G.); stevew@sccwrp.org (S.B.W.); 2 Weston Solutions, 5817 Dryden Place, Suite 101, Carlsbad, CA 92008, USA; Alexander.Schriewer@WestonSolutions.com; 3 City of Malibu, 23825 Stuart Ranch Road, Malibu, CA 90265, USA; asheldon@malibucity.org; 4 Orange County Department of Public Works, 2301 N. Glassell St, Orange, CA 92865, USA; Chris.Crompton@ocpw.ocgov.com; 5 Los Angeles County Department of Public Works, 900 S. Fremont Ave, Alhambra, CA 91803, USA; gamenu@dpw.lacounty.gov; 6 Los Angeles County Sanitation District, 24501 S. Figueroa Street, Carson, CA 90745, USA; jgregory@lacsd.org; 7 Orange County Public Health Laboratory, 600 Shellmaker Road, Newport Beach, CA 92660, USA; JGuzman@ochca.com; 8 National Oceanic and Atmosphere Administration, Ocean Chemistry and Ecosystems Division, Atlantic Oceanographic and Meteorological Laboratory, Miami, FL 33149, stationed at NOAA/NMFS/SWFSC, La Jolla, CA, USA; kelly.goodwin@noaa.gov; 9 City of San Diego Public Utilities Department, Environmental Monitoring and Technical Services Division, 2392 Kincaid Road, San Diego, CA 92101, USA; LOthman@sandiego.gov; 10 City of Encinitas, 505 S. Vulcan Ave, Encinitas, CA 92024, USA; MManasjan@encinitasca.gov; 11 Orange County Sanitation District, 10844 Ellis Ave, Fountain Valley, CA 92708, USA; SChoi@OCSD.COM; 12 Los Angeles Regional Water Quality Control Board, 320 W. 4th St, Suite 200, Los Angeles, CA 90013, USA; Shana.Rapoport@waterboards.ca.gov; 13 Ventura County Public Health Laboratory, 2240 E. Gonzales Road, Oxnard, CA 93036, USA; Syreeta.Steele@sdcounty.ca.gov; 14 City of Los Angeles Environmental Monitoring Division, 12000 Vista del Mar Blvd, Playa del Rey, CA 90293, USA; tommy.nguyen@lacity.org; 15 San Diego Regional Water Quality Control Board, 2375 Northside Dr., Suite 100, San Diego, CA 92108, USA; Helen.Yu@waterboards.ca.gov

**Keywords:** fecal source identification, human fecal marker, storm water, microbial source tracking, water quality monitoring, regional monitoring program, qPCR

## Abstract

Host-associated genetic markers that allow for fecal source identification have been used extensively as a diagnostic tool to determine fecal sources within watersheds, but have not been used in routine monitoring to prioritize remediation actions among watersheds. Here, we present a regional assessment of human marker prevalence among drainages that discharge to the U.S. southern California coast. Approximately 50 samples were analyzed for the HF183 human marker from each of 22 southern California coastal drainages under summer dry weather conditions, and another 50 samples were targeted from each of 23 drainages during wet weather. The HF183 marker was ubiquitous, detected in all but two sites in dry weather and at all sites during wet weather. However, there was considerable difference in the extent of human fecal contamination among sites. Similar site ranking was produced regardless of whether the assessment was based on frequency of HF183 detection or site average HF183 concentration. However, site ranking differed greatly between dry and wet weather. Site ranking also differed greatly when based on enterococci, which do not distinguish between pollution sources, vs. HF183, which distinguishes higher risk human fecal sources from other sources, indicating the additional value of the human-associated marker as a routine monitoring tool.

## 1. Introduction

Recreational water quality is routinely monitored using fecal indicator bacteria (FIB), such as *Enterococcus* spp. and *Escherichia coli*, as proxies for fecal contamination, because they can be measured more economically and quickly than pathogens [1]. Water bodies with FIB concentrations exceeding recreational water quality criteria [2] are treated as a public health risk, and management actions such as beach advisories and pollution remediation are typically implemented in response. 

However, FIB measurements are not diagnostic of whether fecal contamination originates from human, animal or non-fecal sources, which is important for two reasons. First, understanding the sources of fecal contamination allows managers to more appropriately target remediation actions [1,3,4,5]. Second, human fecal material is generally considered a greater public health risk than non-human fecal material [6], making it appropriate to prioritize sites for remediation based on the extent of human fecal contamination. In recognition of different risks posed by different sources, the U.S. Environmental Protection Agency has developed a Quantitative Microbial Risk Assessment (QMRA) process for defining alternative management strategies for beaches that have high FIB counts, but with a corresponding low-level of human fecal contamination [2].

Host-associated genetic markers that allow for fecal source identification and that are sensitive and specific to their target hosts are available [7,8,9,10]. These markers have been used extensively as diagnostic tools to discern fecal sources within watersheds [4,11,12], but have not been used as a monitoring tool to prioritize the need for remediation among watershed systems on a regional scale. Cao et al. [13] identified several challenges in prioritization, such as determining whether the frequency of human marker occurrence or the magnitude of the signal is more important to the outcome. Here, we present a study in which we conduct a regional evaluation of human marker prevalence among drainages that discharge to the southern California Ocean, evaluate the sensitivity of site rankings to some of the decisions outlined by Cao et al. [13] and investigate how those relationships change between periods with, and without, rainfall.

## 2. Materials and Methods

### 2.1. Sites and Sampling

**Figure 1:**
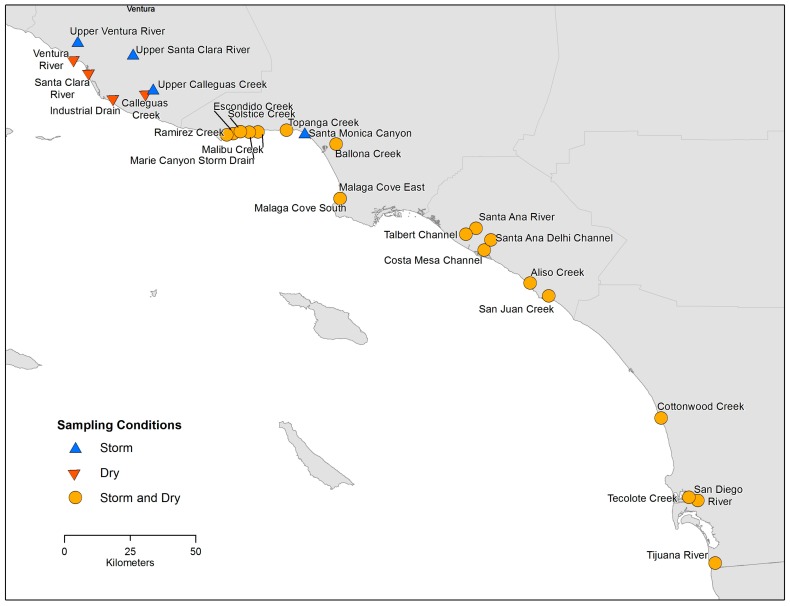
Sampling sites. The majority of the sites were sampled in both storm (i.e., wet weather) and summer dry conditions. An interactive map, including site photos, is available at http://bit.ly/2sxLHcI.

Approximately fifty water samples were collected from each of the twenty-two southern California coastal drainages, which included creeks, rivers and storm drains (Figure 1). Drainages were selected largely based on frequent historical *Enterococcus* spp. exceedances at nearby swimming beaches for which they were an important source water. Samples were collected at approximately weekly intervals under summer dry-weather conditions between 26 June 2013 and 31 October 2015. Some drainages dried out in late summer, leading to a lower number of samples collected. Note that in this study, summer refers to the main recreational season (1 April to 31 October) specified in the California statute (Assembly Bill No. 411). A target of 50 wet weather samples was also collected from each of the 23 drainages (Figure 1) year-round between 1 November 2013 and 26 May 2016, though the number of wet weather samples varied among drainages because of differences in rainfall patterns and capacity in some locations to respond to rainfall events. 

A wet weather event was defined as at least 0.10″ of rainfall at the closest rain gauge to the sampling site following an antecedent dry period of three or more days [14]. While wet weather events are generally confined to the fall and winter seasons in southern California, about 17% of the wet weather samples were collected during summer. 

Dry weather samples were collected following the routine sampling schedule (generally weekly) for each site. Samples were collected in the early morning to mirror current monitoring procedures and limit degradation of the bacterial signal due to sunlight exposure. Wet weather samples were collected as soon as possible following the first 0.10″ of rain, but no later than 72 h after initiation of the rain event. All samples were taken inside the drainage into 0.5-L or 1-L acid washed (10% HCl) polypropylene bottles, upstream of tidal influences and any onsite disinfection facilities (e.g., UV or ozonation, if present) and transported on ice to the laboratory for processing. 

### 2.2. Sample Processing and Quantitative Polymerase Chain Reaction Analysis

Samples from all but four sites under dry weather and from all but three sites under wet weather were tested for cultivable *Enterococcus* by EPA Method 1600 or Enterolert (IDEXX, Westbrook, ME, USA). In addition, samples were filtered (0.45-µM pore size, 47-mm diameter polycarbonate filters, 100 mL per filter or until clogging) within 6 h of sample collection following the standard protocol [15]. Filters were stored at −80 °C until DNA extraction using the GeneRite DNA EZ Extraction kit (GeneRite, North Brunswick, NJ, USA), followed by analysis for the HF183 human-associated fecal marker by the duplex HF183/BacR287 qPCR assay as described elsewhere [9] and in Appendix A. Salmon testes DNA was spiked into the lysis buffer during DNA extraction as a sample processing control and measured by the sketa22 qPCR assay [9,15]. 

Eight laboratories processed samples following the same standard operating protocols for qPCR, including identical qPCR 96-well plate setups, with precise positions indicated for standard curves, negative controls and samples. All samples, standards and controls were run with triplicate qPCR reactions. Each HF183 qPCR plate contained one standard curve (6-point, 10-fold dilution, 1 × 10^6^ to 1 copy per reaction), two negative extraction controls (one for each batch of DNA extractions consisting of 11 samples), one no-template control and 22 environmental samples. All qPCR standards were distributed into vials of single-use volumes at a central facility (Southern California Coastal Water Research Project Authority) and stored at −80 °C before use at the individual laboratories. 

### 2.3. Data Analysis

Master standard curves (cycle of quantification (Cq) vs. log_10_ concentration) were calculated for each laboratory using regression with an outlier removal procedure (a data point was deemed an outlier if its studentized residual was greater than 3), and the resulting regression equations were used for HF183 qPCR quantification as described elsewhere [16]. The limit of detection (LOD) was set at the lowest concentration on the standard curve (1 copy per reaction, >90% replicates amplified at all labs) and expressed in Cq for each lab [17]. The lower limit of quantification (LLOQ) was set at the lowest concentration where all qPCR replicates amplified in all labs (10 copies per reaction) and expressed as Cq + 2 × standard deviation for each lab [17].

Frequencies of HF183 positives and site average HF183 concentrations were calculated as metrics of human fecal contamination at the sites. Samples were defined as positives for frequency calculation if any of the qPCR technical replicates (*n* = 3) amplified. Site average concentrations of HF183 were calculated as the arithmetic mean at the log_10_ scale (i.e., the geometric mean at the normal scale) of all qPCR replicates from all samples at each site. In calculating the means, non-detected (ND) and detection below LOD (DBLOD) [17] were not assigned a zero value, but instead substituted with an estimate based on a Poisson distribution (method detail in Appendix A). Alternative calculations of frequency and concentration were also examined (see Appendix A and Supplementary Materials).

## 3. Results

### 3.1. Sampling and Results Summary

A total of 1013 and 627 samples across all sites from dry and wet weather, respectively, were analyzed for the HF183 human fecal marker. Number of samples per site ranged from 15 to 54 under dry weather and from 3 to 50 under wet weather (see Supplementary Table S1). Sites (none in dry weather, but five in wet weather) with ten or fewer samples were excluded from analyses. 

Standard curves showed satisfactory performance across all labs, with R^2^ and amplification efficiency ranging from 0.95 to 0.99 and from 0.89 to 0.99, respectively (see Table S2). The limit of detection and the lower limit of quantification were 1 and 10 gene copies per reaction, respectively, corresponding to 79 and 789 copies per 100 mL, respectively. Negative controls indicated the absence of contaminant HF183/BacR287 targets in 99.8% of all reactions (2 amplifications (Cq > 37) out of 936 no-template control reactions and 2 amplifications (Cq > 36) out of 1179 negative extraction control reactions). No drainage water samples showed signs of inhibition or sample processing failure. 

The HF183 human fecal marker was detected at all except two sites in dry weather (20 out of 22 sites) and at all sites in wet weather (23 out of 23 sites). Overall, HF183 was detected in 21% and 52% of qPCR reactions in dry and wet weather, respectively. Among the detections, 22% and 44% in dry and wet weather, respectively, were at high enough concentrations to be quantifiable (i.e., above LLOQ). The median HF183 concentration was below LOD in dry weather and at LOD in wet weather. The highest concentrations detected were 1.5 × 10^7^ and 3.2 × 10^6^ copies per 100 mL in dry and wet weather, respectively. Summaries by site are provided in the Supplementary Materials (Figures S1 and S2).

Across all sites, the average *Enterococcus* spp. concentration (geomean) was 237 and 1265 per 100 mL in dry and wet weather, respectively. *Enterococcus* spp. concentration in drainages discharging to the ocean exceeded the California Ocean Plan single sample maximum (SSM) of 104/100 mL for ocean samples in 67% and 88% of samples and was 100-times higher than the SSM in 3% and 19% of samples, in dry and wet weather, respectively (see Table S1). Although the recreational water SSM may not apply to drainage samples, it helps to inform the level of dilution necessary for receiving waters to meet the criteria.

### 3.2. Site Prioritization

**Figure 2:**
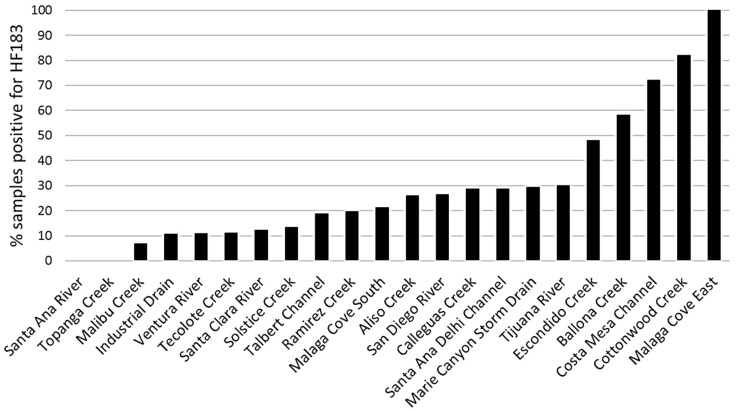
Frequency of HF183 detection at the 22 sites in summer dry weather. Frequency of HF183 detection is defined as % samples that are positive for HF183, and a sample is deemed HF183 positive if HF183 amplified in any of the three qPCR replicates.

Site prioritization was conducted using both frequencies and site average concentrations of HF183 detection, in both dry and wet weather. There were clear differences in the occurrence of the HF183 marker across southern California creeks (Figure 2). Among the 22 sites in dry weather, the marker was not detected in any sample at the two sites (Santa Ana River, Topanga Creek, CA, USA), but was detected in 100% of samples at one site (Malaga Cove East). The frequencies of HF183 detection ranged from 7% to 30% for fifteen sites and from 48% to 100% for five sites. Site average concentrations, ranging from 1 to 154 copies per 100 mL, also showed large site differences (see Figure S2).

**Figure 3:**
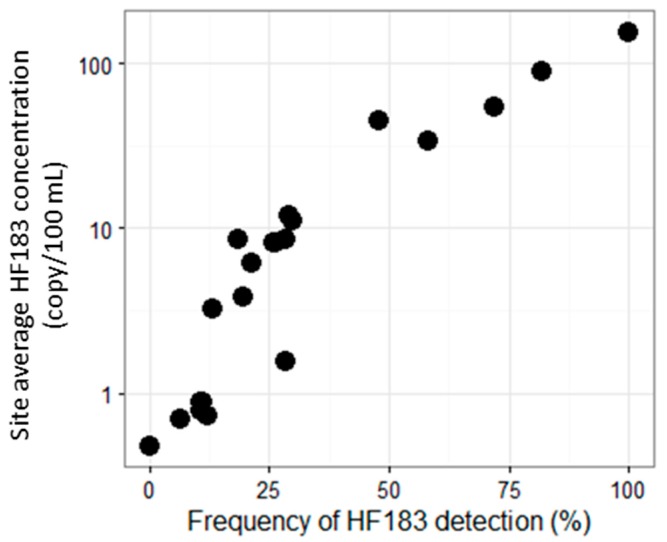
Site average HF183 concentration versus frequency of HF183 detection in summer dry conditions. Frequency of HF183 detection is defined as % samples that are positive for HF183, and a sample is considered positive for HF183 if the marker is amplified in any of the three qPCR replicates. The site average concentration is calculated as the geomean of sample concentrations with non-detected (ND) and DBLOD values substituted by the Poisson approach, as described in Appendix A.

The ranking of drainages was fairly consistent regardless of whether the ranking was based on frequencies of HF183 positives or average HF183 concentrations (Figure 3, Table S3). Among the 22 dry weather sites, five sites received different positions in the ranking based on frequency vs. concentration, among which two sites differed only by one position. All five sites were among the middle positions in ranking by either metric. 

**Figure 4:**
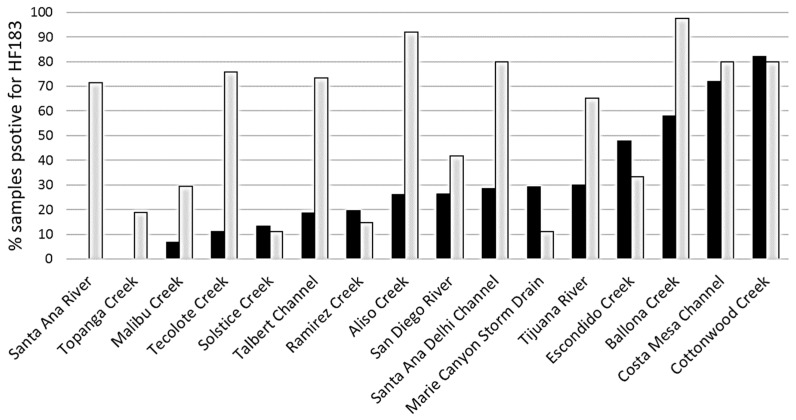
Frequency of HF183 detection by site in wet (light grey filled bars) versus dry (dark-filled) weather conditions. Frequency of HF183 detection is defined as % samples that are positive for HF183 and a sample is deemed positive for HF183 if the HF183 marker amplified in any of the three qPCR replicates. Sites are sorted from left to right by frequency of detection under dry weather conditions.

Site ranking during wet weather did not correlate well with site ranking during dry weather (Figure 4, Figure S3 and Table S4). While the extent of human fecal contamination generally increased (except for three and four sites based on frequency and concentration, respectively, Figure 4 and Figure S3) from dry to wet weather, the relative extent of increase was dissimilar across sites. Among the 16 sites that were sampled in both dry and wet weather, no site had the same rank in dry as rank in wet, regardless of whether the ranking was based on the frequency of HF183 positives or the average site HF183 concentration (see Table S4). Marie Canyon Storm Drain showed the biggest discrepancy, shifting 10 and 11 positions in ranking, based on frequency and concentration, respectively, between dry and wet.

## 4. Discussion

Our study corroborates the ubiquitous presence of human fecal pollution in highly urbanized environments [18,19,20] and is also consistent with the finding of Ahmed et al. [10] that human fecal pollution levels vary considerably across sites. These site differences do not appear to be related to simple watershed characteristics in this study, as we observed no discernable relationship between watershed land use and extent of human fecal contamination in the drainage (see Appendix A). This suggests a complexity of human fecal pollution in urban environments that relates to specific site characteristics and management practices and illustrates the need to conduct site-specific investigations [20]. 

We also observed that the extent of human fecal pollution in drainage systems expands considerably during wet weather events, a finding consistent with previous studies [21]. This is likely caused by increased human fecal input into the drainages during wet weather, perhaps due to overland flow and/or subsurface sewage exfiltration. Depending on storm size and land infiltration capacity in the watershed, storms generate overland flow that brings surrounding fecal pollution into drainages [22]. A raised groundwater table or higher subsurface soil water content can also increase sewage exfiltration as a source of human fecal pollution in drainage systems [23]. However, a few sites showed lower frequencies and concentrations of HF183 in wet than in dry weather, which might be attributed to dilution by the volume of storm water. Regardless, the changes in human fecal signature during rainfall indicate a need to provide evaluation under both wet and dry conditions and suggest that some sites may require different management and remediation strategies between wet and dry periods, with the consideration of flow rate and mass loading.

Site prioritization: One of the study goals was to rank sites in southern California based on the extent of human fecal contamination as a means of prioritizing sites that may require remediation actions. To provide a ranking strategy in absence of any standardized metric, several choices were evaluated. One choice was whether to base site ranking on the frequency of detection or concentration of HF183. For the assay as described here, this choice did not influence ranking appreciably (Table S3), particularly when frequency and site average concentration were defined as in Figure 3. Perhaps this agreement was because sites with persistent human fecal input tended to have higher concentrations, as well, and the calculation of the site average concentration incorporated frequency information (i.e., via a Poisson mean substitution of non-detected (ND) and detected below limit of detection (DBLOD)). While similar rankings were obtained using frequency or concentration in this study, using frequency for site ranking should be easier to implement across laboratories and locations. Sites that warrant further investigation could undergo more detailed analysis, including concentration profiles for the purposes of risk assessment [24]. 

Another choice evaluated was how to define a positive sample for calculating frequency. For the main analysis shown above, we chose to count a sample as positive for HF183 if HF183 amplified, even if the resulting concentration was unquantifiable, in any of the triplicate qPCR reactions. This strategy errs on the side of being more protective of public health as detailed in Appendix A. Motivations for this approach include the recognition that a positive sample at low concentration can produce qPCR replicates that do not amplify due to subsampling effects (see Appendix A) and with DNA extraction loss and PCR inhibition also acting to lower the probability of detection. Although inclusion of any detection can reduce the specificity of the HF183 assay [8], given the wide range of degradation rates of the HF183 marker observed in the environment (see Appendix A), the more protective assumption to include all HF183 signals for site ranking seemed prudent. This strategy also provided the best match to rankings based on the site average concentrations calculated by the Poisson approach. We also tried six other definitions of a positive sample to calculate frequency, deeming a sample positive for HF183 if at least one, or at least two, or all three qPCR replicates amplified, if at least one, or at least two, or all three qPCR replicates were detected, but not quantifiable (DNQ) or quantifiable, or if the sample average concentration (ND and DBLOD values were substituted with ½ or one limit of detection, respectively) was greater than the limit of detection. These alternative frequency calculations were highly correlated across sites (*p*-value < 0.001 for all pair-wise correlation). The rank order of individual sites also did not change appreciably for most alternative frequency definitions (Table S3).

**Figure 5:**
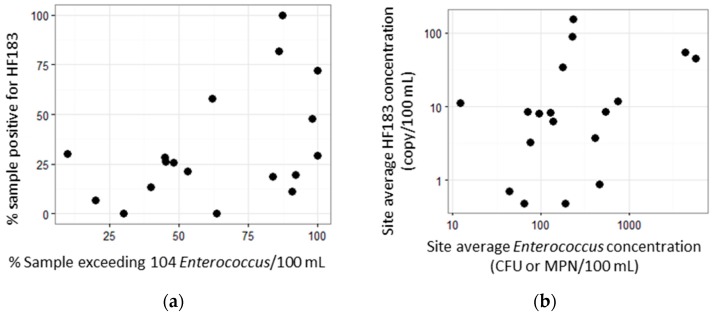
Ranking site by HF183- vs. *Enterococcus*-based metrics during summer dry weather: (**a**) frequency of HF183 detection versus frequency of *Enterococcus* spp. exceedance; (**b**) site average HF183 concentration versus site average *Enterococcus* spp. concentration. HF183-based metrics are defined as in Figure 3. Frequency of *Enterococcus* exceedance is defined as % samples with more than 104 *Enterococcus* per 100 mL. Site average concentration of *Enterococcus* spp. is defined as geomean at the site. CFU and MPN denote colony forming units and most probably number, respectively.

A third choice evaluated for developing site rankings was whether to use HF183 alone or in combination with *Enterococcus* spp., with the rationale that an HF183 signal may be of lesser concern when the *Enterococcus* spp. levels are low. This choice was evaluated by comparing site ranks based on HF183 and site ranks based on *Enterococcus* spp., which were substantially different, as the correlation between ranking by HF183 and ranking by *Enterococcus* spp. was low (Figure 5 and Figure S4). The low correlation probably results because *Enterococcus* spp. is a general indicator that originates from human, non-human [25] and even non-fecal sources [26,27,28,29]. *Enterococcus* spp. and HF183 should correlate only when human fecal material is the dominant source of contamination [30]. As such, we suggest placing higher priority on site ranks based on HF183 than on *Enterococcus* spp. level, with some important special considerations. HF183 is a molecular measurement and its reduction in disinfected water can be much lower than the reduction of culturable *Enterococcus* spp., although the reduction of culturable *Enterococcus* spp. could also be much lower than the reduction of pathogens, depending on the disinfection procedure. Watersheds where recycled water is used for irrigation, for example on golf courses, may warrant further investigations. Additionally, while we focused on site remediation prioritization based on the extent of human fecal contamination, cattle fecal pollution (although not widespread in the southern California region) can pose high public health risk, as well [6].

Implications for water quality management: The utility of the HF183 human fecal marker as a monitoring tool is both valuable and feasible. As FIB do not distinguish between human, non-human or non-fecal sources, monitoring and remediation focused on FIB solely may not be cost-effective if the ultimate goal is public health protection. A monitoring framework that incorporates a more precise fecal indicator such as a human fecal marker should provide a higher level of information and improve management decision making (this study, [20]). Additionally, the HF183 marker assay is becoming widely available for use, with the U.S. EPA presently developing a nationally-standardized analytical procedure [31]. Moreover, a single molecular assay that simultaneously quantifies both *Enterococcus* spp. and the HF183 marker has also been validated [32,33] with published standard operating procedures and multi-media training materials [34]. Our study suggests that routine measurement of both enterococci and the HF183 marker will allow managers to focus remediation efforts on the highest priority sites: those with both high enterococci and HF183 (e.g., upper right corner of Figure 5a).

## 5. Conclusions

- The human fecal-associated HF183 marker was ubiquitous in drainages that discharge into the U.S. southern California coast, but the frequency and magnitude of detection differed greatly across sites.
- Site prioritization for remediation was relatively consistent between prioritization based on frequency of HF183 detection and prioritization based on site average concentration of HF183 detection.
- Compared to dry weather conditions, the extent of human fecal pollution expanded considerably during wet weather events, and site prioritization outcomes also differed under wet weather. These results suggest site ranking is needed for both wet and dry conditions and that different management remediation strategies may be needed to address pollution sources during dry and wet weather time periods.
- Site prioritization based on enterococci, which do not distinguish between pollution sources, differed from site prioritization based on the HF183 marker, which does distinguish higher risk human fecal source from other lower risk sources, indicating the added value of the human fecal-associated marker.
- This study provided a valuable assessment of the status quo of human fecal marker prevalence in a highly urbanized environment and demonstrated the utility of the HF183 marker as a routine monitoring tool.

## Figures and Tables

**Figure A1:**
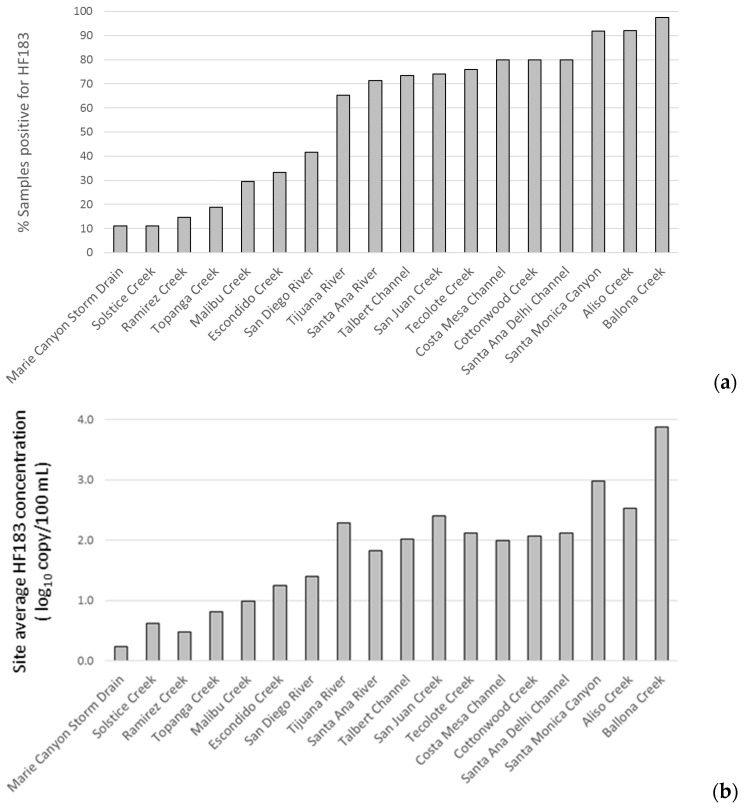
Frequencies of HF183 detection (**a**) and site average HF183 concentrations (**b**) at the 18 sites (number of samples per site >10) during wet weather. The frequency of HF183 detection is defined as % samples that are positive for HF183, and a sample is deemed HF183 positive if HF183 amplified in any of the three qPCR replicates. The site average is calculated by the Poisson substitution approach as described above in Appendix A. Sites are sorted from left to right by the frequency of HF183 positives.

**Figure A2:**
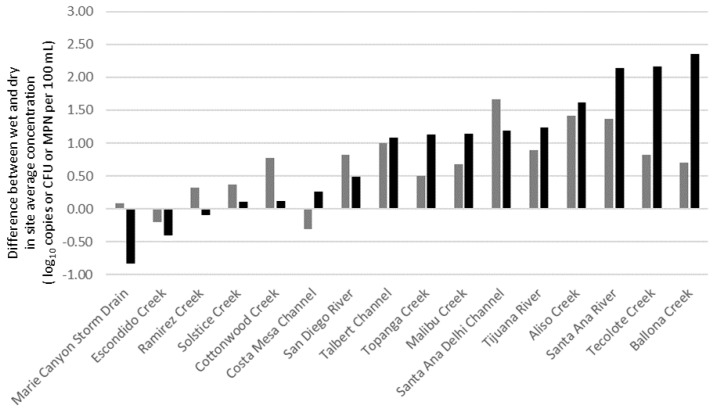
Site average concentration (in log_10_ unit) in wet weather subtracted by that in dry weather for HF183 (black bars) and for enterococci (gray bars). The HF183 site average concentration was calculated by the Poisson approach as described above in the Appendix A. The enterococci site average concentration was calculated directly as the geomean. Sites are sorted from left to right by wet vs. dry differences in site average HF183 concentrations.

**Figure A3:**
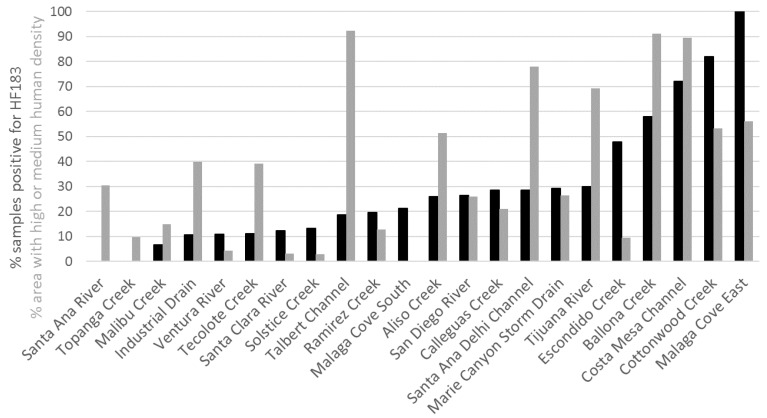
Frequency of HF183 detection in the drainages during dry weather conditions (black bars) and percentage area with high or medium human density in the watershed where the drainage is located (gray bars). Frequency of HF183 detection is defined as % samples that are positive for HF183, and a sample is deemed HF183 positive if HF183 is amplified in any of the three qPCR replicates. Sites are sorted from left to right by frequency of HF183 positives.

**Figure A4:**
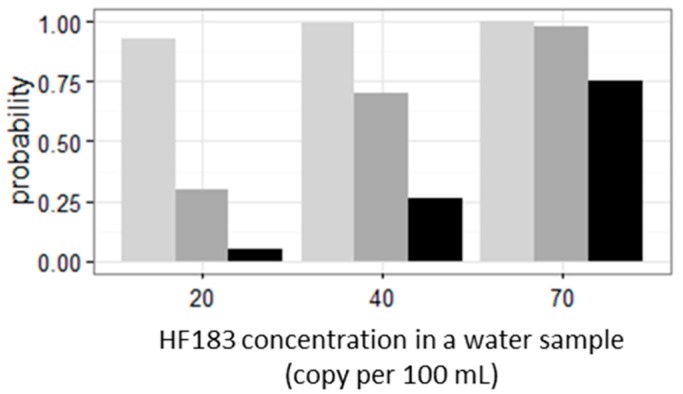
Probability of HF183 amplifying in at least one (light grey), at least two (dark grey) or all three (black) qPCR replicates at low HF183 concentration (*x*-axis). This simulation only accounts for subsampling effects as 2 µL of the 100 µL total DNA extract from the water sample are analyzed in each qPCR reaction, while assuming 100% recovery during DNA extraction and perfect qPCR kinetics (i.e., 100% amplification for all reactions having at least one copy of the target present). Such an assumption is untrue [39], and the real probability of amplification is lower than shown here.

## Supplementary Materials

The following are available online at www.mdpi.com/1660-4601/14/8/874/s1, Figure S1: HF183 results distribution for dry and wet weather; Figure S2: Site average concentration in log10 copies per 100 mL by site for summer dry weather; Figure S3: Site average HF183 concentration by site in wet versus dry weather conditions; Figure S4: Ranking site by HF183-based metrics vs. *Enterococcus*-based metrics during wet weather; Table S1: Sampling summary; Table S2: the HF183 qPCR standard curve parameters and lower limit of quantification; Table S3: Comparison of site ranking based on seven different definitions of the frequency of HF183 positive and two different calculations of site average HF183 concentration; Table S4: Contrast of site ranking positions under wet vs. dry weather.

## Appendix A

The HF183/Bac287 qPCR assay: The 25-µL duplex reaction mixtures contained 1× TaqMan environmental master mix (Version 2.0), 0.2 mg/mL bovine serum albumin (Sigma-Aldrich Corp., St. Louis, MO, USA), 1 µM each primer, 80 nM 6-carboxyfluorescein (FAM)-labeled probe, 80 nM VIC-labeled probe and 2 µL of DNA template or molecular-grade water. The thermal cycling conditions were 10 min at 95 °C followed by 40 cycles of 15 s at 95 °C and 60 s at 60 °C. Sequences of oligonucleotides are reported in Table A1.

**Table A1:** Sequences of primers, probes and standards [9].

Oligonucleotide	Name	Sequence (5′ → 3′)
forward primer	HF183	ATCATGAGTTCACATGTCCG
reverse primer	BacR287	CTTCCTCTCAGAACCCCTATCC
HF183 probe	BacP234MGB	FAM-CTAATGGAACGCATCCC-MGB
IAC probe	BacP234IAC	VIC-AACACGCCGTTGCTACA-MGB
HF183 standard	Standard	ATCATGAGTTCACATGTCCGCATGATTAAAGGTATTTTCCGGTAGACGATGGGGATGCGTTCCATTAGCTCGAGATAGTAGGCGGGGTAACGGCCCACCTAGTCAACGATGGATAGGGGTTCTGAGAGGAAGGTCCCCCACATTGGAACTGAGACACGGTCCAAACTCCTACG
IAC standard	HF183/BacR287 IAC	ATCATGAGTTCACATGTCCGCATGATTAAAGGTATTTTCCGGTAGACGATGTGTAGCAACGGCGTGTTATAGTAGGCGGGGTAACGGCCCACCTAGTCAACGATGGATAGGGGTTCTGAGAGGAAG

Calculating site average HF183 concentration: The site average concentration of HF183 was calculated as the arithmetic mean at the log_10_ scale (i.e., the geometric mean at the normal scale) of all qPCR replicates from all samples at each site. Depending on the target concentration, qPCR results were grouped into four categories: ND (non-detected, i.e., no amplification), DBLOD (detected below limit of detection), DNQ (detected, but not quantifiable) and quantifiable [17]. In calculating the means, ND and DBLOD were considered censored data, and DNQ and quantifiable data were used as is. Two site average concentrations were calculated, differing by how the censored data were treated before averaging. Our first approach substituted ND and DBLOD with ½ or one limit of detection, respectively, similar to substitution strategies used for certain chemical analysis. The effect of substituting ND with Cq = 40 [8] was not evaluated here. Although, such simple substitution strategies have been heavily criticized, certain statistically-based alternatives call for <80% censored data [35]. If a prerequisite of <80% censored data and >10 uncensored data points (including qPCR technical replicates) were applied, the majority of the sites (17 out of 22 in summer dry) would need to be excluded; therefore, such alternative approaches were not reported here. Our second approach substituted samples with ND and DBLOD with a Poisson mean estimated by regarding ND as negative and DBLOD as positive. The fraction of positives (p) was then used to estimate a Poisson mean λ = −ln(1 − p), based on the total number of ND and DBLOD reactions per site (see Supplementary Materials Figure S1a).

HF183 results in wet weather: Sixteen sites were sampled in both dry and wet weather, and a total of 18 sites sampled during wet weather had more than 10 samples. Large differences in the extent of the prevalence of the HF183 marker was observed across the 18 sites (Figure A1). The HF183 marker was detected in 11% to 97% of samples, and site average HF183 concentration ranged from two to 7551 copies per 100 mL.

*Enterococcus* spp. levels in dry versus wet weather: Similar to the dry versus wet contrast for HF183, the extent of enterococci levels (% samples exceeding 104 enterococci/100 mL or enterococci site geomean) generally increased (except for two sites) from dry to wet weather, and the relative extent of increase was dissimilar across sites. Among the 16 sites sampled in both dry and wet weather, the changes in site average concentrations from dry to wet for enterococci and for HF183 did not track each other exactly across sites (Figure A2), but the overall trend in the dry-to-wet changes was significantly correlated between enterococci and HF183 (*p* < 0.05, R^2^ = 0.44, *n* = 16).

Characterization of watershed land use pattern: To discern potential drivers of the observed large site differences in the level of human fecal pollution, watershed land use patterns were correlated to the extent of human fecal contamination in drainages. The land use patterns were characterized by arranging land use categories (provided by responsible agencies for the studied watersheds based on the analysis of GIS land use profiles) into four bins roughly based on the density of human occupancy, as follows. High human density included single family and multi-family residential areas; medium human density included commercial, institutional, municipal and office areas; low human density included areas with education industrial and other similar facilities; minimal human density included agriculture, open space, parks, transportation, vacant, water and similar areas. The percentage area in each bin (and with bins of high and medium human density combined) was then correlated to the frequency of HF183 detection and the site average concentration of HF183. No significant correlation was observed (Figure A3). Although the four sites with the highest frequency and average concentration of HF183 detection had more than 50% of the watershed characterized as having relatively high human occupancy (commercial and/or residential), other watersheds with similar land use had much less human fecal contamination (<30% HF183 detection). Characterizations with regard to infrastructure age and geological conditions were not assessed.

Rational for the definition of an HF183 positive sample: To calculate the frequency of HF183 detection, a sample was deemed positive (for HF183 detection) if this marker amplified in any of the three qPCR replicates. This method errs on the side of being more protective of public health and is based primarily on two lines of reasoning, as detailed below: (1) the possibility that a low HF183 signal could nonetheless warrant further investigation; and (2) not all qPCR replicates will amplify when the HF183 signals are low.

In diluted fresh sewage, 4200 copies HF183 per 100 mL corresponds to a U.S. EPA benchmark illness rate of public health significance (30 GI illnesses per 1000 swimmers) based on quantitative microbial risk assessment analysis of fresh sewage [36]. However, environmental waters mostly contain aged sewage or septage with a wide range of reported decay rates [10] that depend on pollution source and environmental conditions. Recent field in situ experiments in California measured decay rates corresponding to a 0.5 to 3 log_10_ reduction per day, leading to a reduction of the 4200 copies of HF183 per 100 mL to below or near the limit of detection of qPCR within one to three days and found that the HF183 marker can decay faster than pathogens under environmental conditions [37]. Although the initial concentration of the HF183 marker is expected to be much higher compared to initial pathogen concentrations [38], the possibility that low HF183 signals could represent a need for remediation actions motivated the inclusion of all HF183 signals for site prioritization. More detailed fate and transport and QMRA analysis may be performed for the purposes of risk assessment [24].

A primary motivation for counting a sample as positive if any triplicate reaction is positive is that only a small fraction (<2%) of the original water sample is analyzed based on the HF183 protocol [9] (and in most qPCR protocols). A simulation demonstrates that low HF183 signals may only be detected in one out of the three qPCR replicates due to subsampling effects (Figure A4). Briefly, generally, 100 mL of water are filtered to capture bacterial cells on the filter, which is subject to DNA extraction resulting in 100 µL DNA extract, from which only 2 µL were analyzed in each qPCR reaction to measure the HF183 marker. A subsampling process therefore occurs when pipetting 2 µL out of the 100 µL total DNA volume, and this process reduces the probability of detection when the HF183 concentration is low. While other factors such as DNA extraction efficiency and PCR kinetics further reduce the probability of amplification, a simulation using a binomial distribution can be used to demonstrate how subsampling (of the DNA extracts) alone reduces the probability of amplification in qPCR replicates.

A binomial distribution of B(3, p.rxn) was used to estimate the probability of observing HF183 amplification in at least one, or at least two, or in all three qPCR replicates, where p.rxn = the probability of having at least one copy of the HF183 marker present in each qPCR reaction. p.rxn was calculated by simulating the subsampling process of pipetting 2 µL of template (per qPCR standard operating protocol) from the 100 µL DNA extracts (resulting from 100 mL of water) into each qPCR reaction. The subsampling process was simulated by B(2, conc) where 2 refers to the 2 µL DNA extracts added to each qPCR reaction, and conc = HF183 copies/µL DNA extracts (i.e., copies of HF183/µL DNA extract is equivalent to HF183/mL of water sample, assuming 100% recovery during DNA extraction).
