# Supplementary material for: Regional Assessment of Human Fecal Contamination in Southern California Coastal Drainages
Source: Int J Environ Res Public Health. 2017 Aug 4;14(8):874. doi: 10.3390/ijerph14080874 (PMC5580578; doi:10.3390/ijerph14080874)
Supplement: Supplementary file 1 [file ijerph-14-00874-s001.pdf]

International Journal of
Environmental Research and Public Health

MDPI

Supplementary Materials

Regional Assessment of Human Fecal Contamination in Southern California Coastal Drainages

Yiping Cao, Meredith R. Raith, Paul D. Smith, John F. Griffith, Stephen B. Weisberg, Alexander Schriewer, Andrew Sheldon, Chris Crompton, Geremew G. Amenu, Jason Gregory, Joe Guzman, Kelly D. Goodwin, Laila Othman, Mayela Manasjan, Samuel Choi, Shana Rapoport, Syreeta Steele, Tommy Nguyen, Xueyuan Yu

Table S1. Sampling summary. Sites are listed approximately from north to south as shown in the map. Column headings n.HF183, n.Ent, %>104, %>10,400 refer to the number of samples analyzed for HF183, the number of samples analyzed for *Enterococcus* spp., % of samples with greater than 104 *Enterococcus* per 100 mL and % of samples with greater than 10,400 *Enterococcus* per 100 mL. Some samples were not analyzed for enterococci due to lab errors.

	**Summer dry**				**Wet**			
**Site**	**n.HF183**	**n.Ent**	**% >104**	**% >10,400**	**n.HF183**	**n.Ent**	**% >104**	**% >10,400**
Upper Ventura River	-	-	-	-	10	-	-	-
Ventura River	46	-	-	-	-	-	-	-
Upper Santa Clara River	-	-	-	-	7	-	-	-
Santa Clara River	49	-	-	-	-	-	-	-
Industrial Drain	47	-	-	-	-	-	-	-
Upper Calleguas Creek	-	-	-	-	8	-	-	-
Calleguas Creek	49	-	-	-	-	-	-	-
Ramirez Creek	51	51	92	0	34	34	94	0
Escondido Creek	48	48	98	29	36	36	97	25
Solstice Creek	30	30	40	0	27	27	70	0
Marie Canyon Storm Drain	48	48	100	2	36	36	100	0
Malibu Creek	15	15	20	0	34	34	65	0
Topanga Creek	43	43	30	0	32	32	69	0
Santa Monica Canyon	-	-	-	-	37	37	100	24
Ballona Creek	50	50	62	0	39	39	74	13
Malaga Cove South	47	47	53	0	3	3	100	0
Malaga Cove East	47	47	87	0	4	4	100	0
Talbert Channel	43	43	84	2	15	15	100	33
Santa Ana River	44	44	64	0	14	14	93	64
Santa Ana Delhi Channel	49	49	45	0	50	50	98	38
Costa Mesa Channel	50	50	100	10	50	50	86	32
Aliso Creek	50	50	48	0	50	50	98	18
San Juan Creek	-	-	-	-	50	50	94	32
Cottonwood Creek	50	50	86	2	15	15	87	27
Tecolote Creek	54	54	91	2	29	27	100	33
San Diego River	53	53	45	4	24	24	92	8
Tijuana River	50	50	10	2	23	22	55	5
Overall	1013	822	67	3	627	599	88	19

Table S2. The HF183 qPCR standard curve parameters and lower limit of quantification (LLOQ) in Cq. E is the qPCR amplification efficiency $E = 10^{-1/slope}-1$. Column heading n indicates number of data points available for calculating the master standard curves.

Lab	Slope	Y intercept	R^2	n	E	LLOQ in Cq
1	−3.52	38.98	0.993	172	0.92	35.5
2	−3.42	36.32	0.983	231	0.96	32.9
3	−3.38	35.06	0.994	190	0.97	31.7
4	−3.49	36.64	0.987	290	0.94	33.2
5	−3.35	36.76	0.966	107	0.99	33.4
6	−3.51	38.43	0.988	372	0.93	34.9
7	−3.57	39.23	0.952	178	0.91	35.7
8	−3.63	38.89	0.988	83	0.89	35.3

Table S3. Comparison of site ranking based on seven different definitions of the frequency of HF183 positive (amp1, amp2, amp3, lod1, lod2, lod3, lodAvg) and two different calculations of site average HF183 concentration (AvgPois, AvgLod), in dry (A) and wet (B) weather. The frequency of HF183 positives was defined as the percentage of samples that were positive for HF183 at each site. Seven different definitions of a positive sample were used to calculate frequency. A sample was deemed positive for HF183 if at least one (amp1), or at least two (amp2), or all three (amp3) qPCR replicates amplified; if at least one (lod1), or at least two (lod2), or all three (lod3) qPCR replicates were detected, but not quantifiable (DNQ) or quantifiable; or if the sample average concentration was greater than limit of detection (lodAvg). In calculating the sample average for determining lodAvg, ND and detected below limit of detection (DBLOD) were substituted with ½ or 1 limit of detection. Site average HF183 concentration was calculated by substituting ND and NBLOD with a statistically-based Poisson estimate (AvgPois) or with ½ or 1 limit of detection, respectively (AvgLod).

**(A) Dry weather**		**Site ranking position**								
**Site[2]**	**n.spl[1]**	**amp1**	**amp2**	**amp3**	**lod1**	**lod2**	**lod3**	**lodAvg**	**AvgPois**	**AvgLod**
Santa Ana River	44	**1.5**	1.5	2	1.5	1.5	2	1.5	**1.5**	1.5
Topanga Creek	43	**1.5**	1.5	2	1.5	1.5	2	1.5	**1.5**	1.5
Malibu Creek	15	**3**	9	13	6	13	16	10	**3**	3
Industrial Drain	47	**4**	5	9	9	7.5	10.5	7	**5**	6
Ventura River	46	**5**	11	10	10	14	12	13	**6**	9
Tecolote Creek	54	**6**	6	4	8	10	4	12	**7**	11
Santa Clara River	49	**7**	4	2	11	5.5	2	5.5	**4**	5
Solstice Creek	30	**8**	3	6	3	3	6	3	**9**	4
Talbert Channel	43	**9**	14	14	7	9	13	11	**14**	10
Ramirez Creek	51	**10**	13	11	16	11	7	8	**10**	12
Malaga Cove South	47	**11**	8	12	12	7.5	10.5	9	**11**	13
Aliso Creek	50	**12**	10	7	4	4	8	4	**12**	7
San Diego River	53	**13**	16	15	15	16	14	16	**13**	16
Santa Ana Delhi Channel	49	**14.5**	7	8	5	5.5	9	5.5	**15**	8
Calleguas Creek	49	**14.5**	12	5	17	15	5	15	**8**	15
Marie Canyon Storm Drain	48	**16**	17	17	14	17	17	17	**17**	17
Tijuana River	50	**17**	15	16	13	12	15	14	**16**	14
Escondido Creek	48	**18**	18	18	18	19	19	18	**19**	20
Ballona Creek	50	19	19	19.5	20	20	20	20	18	19
Costa Mesa Channel	50	20	20	19.5	19	18	18	19	20	18
Cottonwood Creek	50	21	21	21	21	21	21	21	21	21
Malaga Cove East	47	22	22	22	22	22	22	22	22	22

**(B) Wet weather**		**Site ranking position**								
**Site**[2]	**n.spl**[1]	**amp1**	**amp2**	**amp3**	**lod1**	**lod2**	**lod3**	**lodAvg**	**AvgPois**	**AvgLod**
Marie Canyon Storm Drain	36	1.5	2	4	2	4	4	4	1	4
Solstice Creek	27	1.5	1	1.5	1	1	1.5	1.5	3	1
Ramirez Creek	34	3	3	1.5	3	2	1.5	1.5	2	2
Topanga Creek	32	4	4	3	4	3	3	3	4	3
Malibu Creek	34	5	5	6	5	6	5	6	5	5
Escondido Creek	36	6	6	5	6	5	7	5	6	7
San Diego River	24	7	7	7	7	7	6	7	7	6
Tijuana River	23	8	10	10	9	10	11	11	14	14
Santa Ana River	14	9	11	9	10	11	8	8	8	8
Talbert Channel	15	10	13	15	14	13	14	15	10	10
San Juan Creek	50	11	14.5	14	13	14.5	15	14	15	15
Tecolote Creek	29	12	9	12	12	12	13	12	12	13
Costa Mesa Channel	50	14	12	11	11	8	10	9	9	9
Santa Ana Delhi Channel	50	14	14.5	13	15	14.5	12	13	13	12
Cottonwood Creek	15	14	8	8	8	9	9	10	11	11
Santa Monica Canyon	37	16	17	17	17	17	17	17	17	17
Aliso Creek	50	17	16	16	16	16	16	16	16	16
Ballona Creek	39	18	18	18	18	18	18	18	18	18

[1] The number of samples at each site is denoted by n.spl, and sites with 10 or fewer samples are excluded. Each sample had n = 3 qPCR technical replicates; [2] Sites are sorted from lowest to highest ranking position (i.e., having the least to having the most human fecal contamination) based on the "amp1" frequency definition.

Table S4. Site rank contrast under wet vs. dry weather, as ranked by frequency or site average concentration. Sites are sorted from left to right by frequency of detection under dry weather conditions. Frequency of HF183 detection is defined as % samples with HF183 detection, and HF183 is said to be detected in a sample if HF183 is detected in any of the three qPCR replicates. Sites are sorted by ranking positions under dry weather.

	**Rank by frequency**			**Rank by Site average concentration**	
Site	Dry	Wet	Site	Dry	Wet
Santa Ana River	1.5	9	Santa Ana River	1.5	8
Topanga Creek	1.5	4	Topanga Creek	1.5	4
Malibu Creek	3	5	Malibu Creek	3	5
Tecolote Creek	4	11	Tecolote Creek	4	12
Solstice Creek	5	1.5	Solstice Creek	5	3
Talbert Channel	6	10	Ramirez Creek	6	2
Ramirez Creek	7	3	Aliso Creek	7	15
Aliso Creek	8	15	San Diego River	8	7
San Diego River	9	7	Talbert Channel	9	10
Santa Ana Delhi Channel	10	13	Santa Ana Delhi Channel	10	13
Marie Canyon Storm Drain	11	1.5	Tijuana River	11	14
Tijuana River	12	8	Marie Canyon Storm Drain	12	1
Escondido Creek	13	6	Ballona Creek	13	16
Ballona Creek	14	16	Escondido Creek	14	6
Costa Mesa Channel	15	13	Costa Mesa Channel	15	9
Cottonwood Creek	16	13	Cottonwood Creek	16	11

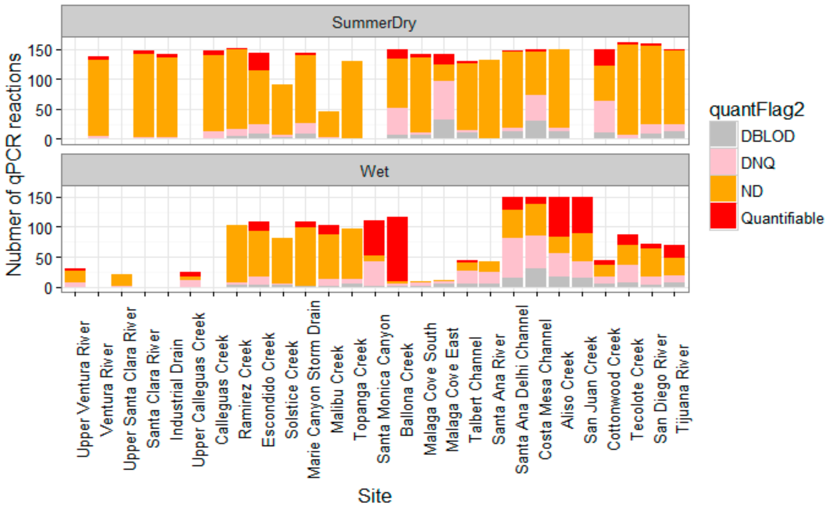

(a)

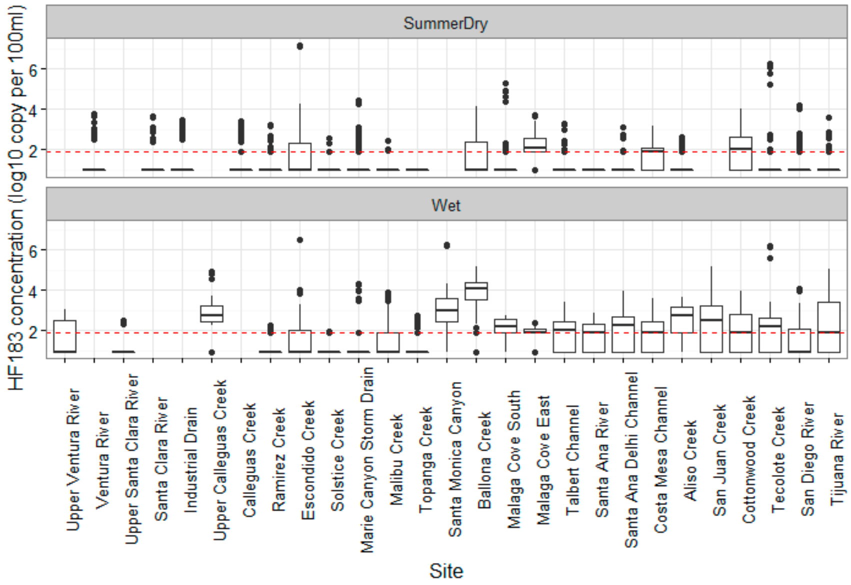

(b)

Figure S1. HF183 results distribution for dry and wet weather: (**a**) number of qPCR results in each quantification category, (**b**) HF183 concentrations (ND and DBLOD substituted with ½ and one of the limit of detection for the purpose of plotting). The dotted red line in (b) indicates the limit of detection. Note that empty spaces along the x-axis indicate that the site was not sampled during the given weather condition.

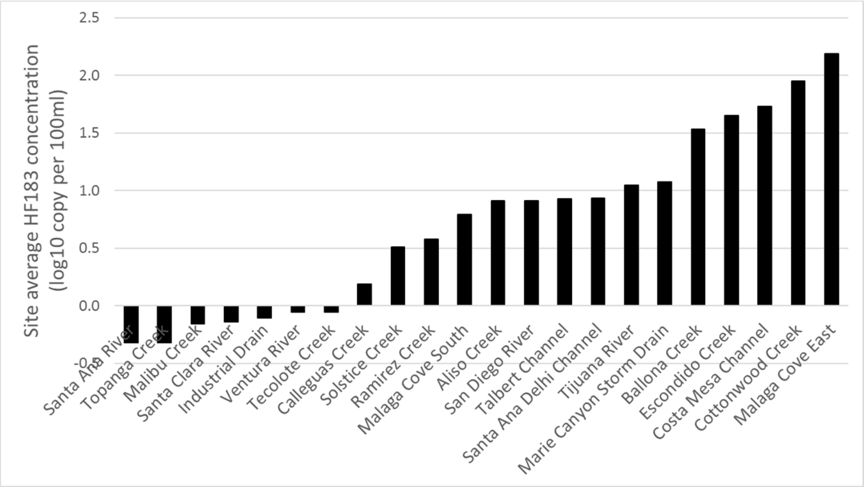

Figure S2. Site average HF183 concentration in log_{10} copies per 100 mL by site for summer dry weather. The site average is calculated by the Poisson approach as described in the Appendix section.

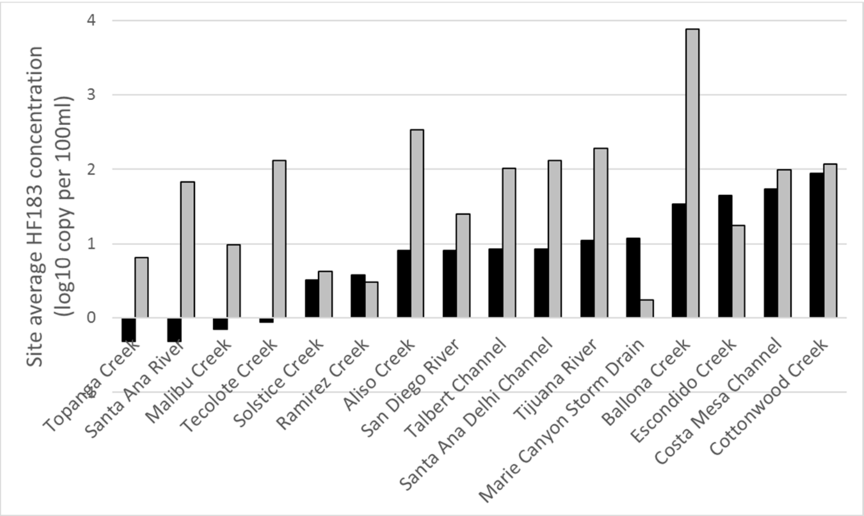

Figure S3. Site average HF183 concentration in log_{10} copies per 100 mL by site in wet (grey bars) versus dry (black bars) weather conditions. The site average is calculated by the Poisson approach as described in the Appendix section. Sites are sorted from left to right by site average concentration under dry weather conditions.

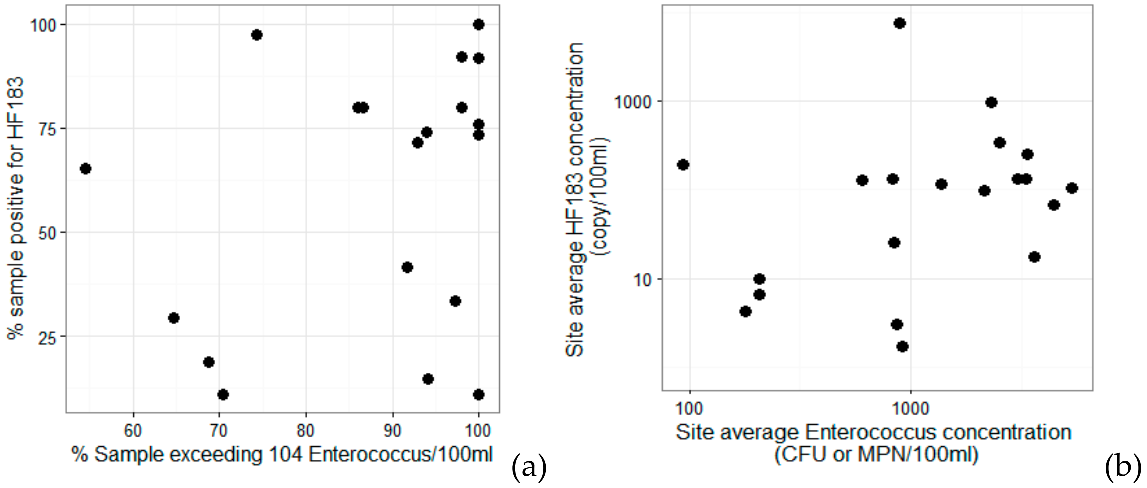

Figure S4. Ranking site by HF183-based metrics vs. *Enterococcus*-based metrics during wet weather: (**a**) Frequency of HF183 positive versus frequency of *Enterococcus* exceedance; (**b**) site average HF183 concentration versus site average *Enterococcus* concentration. HF183-based metrics are as defined in Figure 3. The frequency of *Enterococcus* exceedance is defined as % samples with more than 104 *Enterococcus* spp. per 100 mL. The site average concentration of *Enterococcus* spp. is defined as the geomean at the site.
